# Increased rate of sporadic and recurrent rare genic copy number variants in Parkinson's disease among Ashkenazi Jews

**DOI:** 10.1002/mgg3.18

**Published:** 2013-06-07

**Authors:** Xinmin Liu, Rong Cheng, Xin Ye, Miguel Verbitsky, Sergey Kisselev, Helen Mejia-Santana, Elan D Louis, Lucien J Cote, Howard F Andrews, Cheryl H Waters, Blair Ford, Stanley Fahn, Karen Marder, Joseph H Lee, Lorraine N Clark

**Affiliations:** 1 Taub Institute for Research on Alzheimer's Disease and the Aging Brain, College of Physicians and Surgeons, Columbia UniversityNew York, New York; 2 Gertrude H. Sergievsky Center, College of Physicians and Surgeons, Columbia UniversityNew York, New York; 3 Department of Pathology and Cell Biology, College of Physicians and Surgeons, Columbia UniversityNew York, New York; 4 Department of Neurology, College of Physicians and Surgeons, Columbia UniversityNew York, New York; 5 Department of Epidemiology, Mailman School of Public Health, Columbia UniversityNew York, New York; 6 New York State Psychiatric Institute, Data Coordinating CenterNew York, New York; 7 Department of Psychiatry, Columbia University Medical CenterNew York City, New York; 8 Center for Human Genetics, College of Physicians and Surgeons, Columbia UniversityNew York, New York

**Keywords:** Ashkenazi Jews, candidate genes, case–control study, CNV, Parkinson's disease

## Abstract

To date, only one genome-wide study has assessed the contribution of copy number variants (CNVs) to Parkinson's disease (PD). We conducted a genome-wide scan for CNVs in a case–control dataset of Ashkenazi Jewish (AJ) origin (268 PD cases and 178 controls). Using high-confidence CNVs, we examined the global genome wide burden of large (≥100 kb) and rare (≤1% in the dataset) CNVs between cases and controls. A total of 986 such CNVs were observed in our dataset of 432 subjects. Overall global burden analyses did not reveal significant differences between cases and controls in CNV rate, distribution of deletions or duplications or number of genes affected by CNVs. Overall deletions (total CNV size and ≥2× frequency) were found 1.4 times more often in cases than in controls (*P* = 0.019). The large CNVs (≥500 kb) were also significantly associated with PD (*P* = 0.046, 1.24-fold higher in cases than in controls). Global burden was elevated for rare CNV regions. Specifically, for *OVOS2* on Chr12p11.21, CNVs were observed only in PD cases (*n* = 7) but not in controls (*P* = 0.028) and this was experimentally validated. A total of 81 PD cases carried a rare genic CNV that was absent in controls. Ingenuity pathway analysis (IPA) identified *ATXN3*, *FBXW7*, *CHCHD3*, *HSF1*, *KLC1*, and *MBD3* in the same disease pathway with known PD genes.

## Introduction

Genetic linkage studies of Parkinson's disease (PD) have identified a number of genes which include *SNCA* (*PARK1*) (Polymeropoulos et al. 1997), *Parkin* (*PARK2*) (Kitada et al. 1998), PTEN-induced putative kinase (*PINK1*; *PARK6*) (Valente et al. 2004), *DJ-1* (*PARK7*) (Bonifati et al. 2003b) and leucine rich repeat kinase 2 (*LRRK2*; *PARK8*) (Paisan-Ruiz et al. 2004), *ATP13A2* (*PARK9*) (Ramirez et al. 2006), *PLA2G6* (*PARK14*) (Paisan-Ruiz et al. 2009), *FBXO7* (*PARK15*) (Fonzo et al. 2009), and *VPS35* (*PARK17*) (Vilariño-Güell et al. 2011; Zimprich et al. 2011). Mutations in these genes are rare and highly penetrant with large effects (e.g., *Parkin*, *PARK2*), and their prevalence may vary substantially by age at onset (AAO), family history of PD (FHPD), and ethnicity (Lees et al. 2009; Nuytemans et al. 2010). Common genetic variants defined as variants with a minimum allele frequency (MAF) ≥5% are also believed to contribute to PD disease susceptibility. PD genome-wide association studies (GWAS) in different populations worldwide, in addition to meta-analyses of GWAS datasets, have identified a number of candidate susceptibility loci and genes with odd ratio estimates of 1.2–1.4 (Lill et al. 2012). A recently proposed method based on genome-wide sharing estimates between distantly related individuals, estimated the heritability of late onset PD in a North American population to be at least 0.27 (Do et al. 2011). Yet, genetic variance explained by reported single-nucleotide polymorphisms (SNPs) does not reach the reported heritability estimates, suggesting that a substantial component of the genetic variance in PD remains unexplained (Do et al. 2011). This missing heritability may be explained partly by rare and de novo variants.

Copy number variation (CNV) has been associated with several complex diseases, including neurodegenerative disease (Lee and Lupski 2006). In PD patients, rare large genomic duplications or deletions have also been observed in the PD genes *SNCA* (*PARK1*) (Singleton et al. 2003), *Parkin* (*PARK2*) (Kitada et al. 1998), and *DJ1* (*PARK7*) (Bonifati et al. 2003a) and in a gene that causes dystonia/infantile parkinsonism, tyrosine hydroxylase (*TH*) (Bademci et al. 2010). To date, only one genome-wide case–control study has assessed the contribution of CNVs to familial PD in a North American dataset (Pankratz et al. 2011). In this study, only CNVs within the *PARK2* locus could be molecularly validated and associated with PD at genome wide significance, and no new loci were identified.

To identify novel CNVs and to evaluate the contribution of those CNVs to the risk of PD, we analyzed an Ashkenazi Jewish (AJ) dataset of unrelated cases (*n* = 268) and controls (*n* = 178) with similar age and sex distributions that was previously assessed in a SNP-based GWAS for the role of common variants in PD in an AJ population from NY (Liu et al. 2011). We have focused on a genetic isolate, the AJ population, as a discovery dataset since this cohort has a higher sharing of genetic background, and historically experienced a significant bottleneck. We hypothesized that founder CNVs or risk CNVs shared by multiple AJ PD cases would be identified in this population. In this study we had two main aims. First, we evaluated the genome-wide burden of CNVs (common and rare) to determine whether individuals with PD have a greater genome wide burden of CNVs than unaffected individuals. Second, we assessed the contribution of rare genic CNVs to Parkinson disease association.

## Material and Methods

### Subjects

The AJ GWAS dataset was created by combining participants from two studies, the Genetic Epidemiology of PD study (PD EPI) (Marder et al. 2003) and the AJ Study (Liu et al. 2011). The ascertainment of cases (*n* = 168) and controls (*n* = 84) for the PD EPI study was described in detail in Marder et al. (2003) and the ascertainment of cases (*n* = 100) and controls (*n* = 94) for the AJ study is described below. Briefly, for the PD EPI and AJ study, PD cases were recruited from the Center for PD and Other Movement Disorders at Columbia University. All met research criteria for PD. All controls underwent the same evaluation as cases, which included a medical history, Unified PD Rating Scale (UPDRS) and Mini Mental State Exam (MMSE) (Marder et al. 2003). Family history of PD and related disorders in first-degree relatives was obtained using a structured interview that has been shown to be reliable and valid.

The PD EPI study was enriched for cases with AAO of 50 years of age or younger and the majority of controls were recruited via random digit dialing. Initially, information on Jewish ancestry in each of the grandparents was obtained during an interview. Information about Ashkenazi origin was not specifically obtained; however, ∼90% of Jews in the United States are Ashkenazi. For the AJ study, PD cases were recruited specifically based on their AJ ancestry and information on AJ ancestry in each of the grandparents was obtained during an interview. We subsequently used the GWAS data to assess whether there exists substantial population stratification, and adjusted population clusters in the analysis. Principal component analysis (PCA) comparing eigenvectors in the entire sample (*n* = 446) was also used to assess whether there exist cryptic population subclusters and how closely cases and controls match (Fig. S1). PCA analysis revealed that 14 individuals clustered separately from the rest of the cohort and we adjusted for clustering and PCA in the analysis. In this cohort, the mean Identity-by descent (IBD) sharing for cases did not differ from that for controls (0.01 vs. 0.009, respectively).

We also used genome-wide 99,393 unlinked SNPs to compute individual inbreeding coefficient F to assess heterozygosity for cases and controls, separately (PLINK). These SNPs were selected by looking at a 5-SNP window from a set of 50 SNPs in which variance inflation factor was set at 1.5. The mean inbreeding coefficient for cases was 0.0034 (±0.0127, range 0∼0.1404) while that for controls was controls: 0.0024 (±0.0066, range 0∼0.0487). While these average inbreeding coefficients show cryptic relatedness of third∼fourth cousins, there was no significant difference between cases and controls (*P* = 0.30).

This study was approved by the Institutional Review Board at Columbia University Medical Center. Each study participant signed a written informed consent approved by the University Human Ethics Committee.

### Genotyping and quality control assessment

A total of 268 cases and 178 controls were genotyped using the Illumina (San Diego, CA, USA) Human 610-quad bead arrays (cases *n* = 91 and controls *n* = 96) or the Illumina Human 660-quad bead arrays (cases *n* = 191 and controls *n* = 84). All DNAs were derived from whole blood. *GBA* and *LRRK2* mutation status has been reported previously for all samples (Clark et al. 2006, 2007).

Quality scores were determined from allele cluster definitions for each SNP as determined by the Illumina GenomeStudio Genotyping Module version 3.0 and the combined intensity data from 100% of study samples. Genotype calls with a quality score (Gencall value) of 0.25 or higher were considered acceptable. We genotyped 10 samples in duplicate to assess genotyping accuracy and found blind duplicate reproducibility to be 100%. In addition, 10 cases and two controls were genotyped by the above two platforms, and for the overlapping SNPs, genotypes showed 100% concordance. Six individuals with high IBD sharing were removed. When an individual was genotyped more than once, we used the genotype data from the Illumina Human 660-quad bead arrays.

### Population stratification

We examined ancestry for each subject to estimate cryptic population stratification using the identity-by-state (IBS)-based clustering method as implemented in PLINK (version 1.07) (Purcell et al. 2007). Briefly, we used all available SNPs (*n* = 522,578 autosomal SNPs) for the PLINK analysis to assess underlying population structure. To assess potential cryptic population stratification, we augmented the 446 AJ samples with white subjects from the HapMap website (http://www.hapmap.org/), which included 60 European Americans, 60 Yorubans, and 90 Asians. The best fitting model assumed two underlying populations; however, the proportion of the second cluster was small (*n* = 14), and this group of individuals clustered with the HapMap whites. PCA as implemented in the GCTA package (Yang et al. 2011) was also used to examine ancestry and admixture in the entire sample (*n* = 446) together with 120 HapMap individuals [60 Yoruba in Ibadan Nigeria and 60 CEU (Utah residents with Northern and Western European ancestry from the Centre d'Etude du Polymorphisme Human, CEPH)]. Projection of all the samples genotypes along the first three principal components is shown in Figure S2. Although there is tight clustering of the AJ samples we do observe overlap of 14 individuals with the CEU cluster as we did previously in our analysis using IBS-based clustering. We adjusted for the second population cluster by including population cluster as a covariate in the analysis.

### Quality control assessment for CNV

Raw intensity array data were normalized within and across samples using Illumina's GenomeStudio software. The CNV calls were generated by PennCNV (version 2011 June) (Wang et al. 2007) using the log R ratio (LRR) and B allele frequency (BAF) measures automatically computed from the signal intensity files of GenomeStudio, and the standard hg18 “all” PennCNV hidden Markov model (hmm) and population frequency of B allele (pfb) files for the 610 and 660 BeadChips.

Extensive QC protocols were applied, blinded to PD affection status, and poor quality experiments were excluded from the study, if they met the following criteria: array call rate <99.8%, log R ratio standard deviation >0.27, B allele frequency standard deviation >0.17 and PennCNV wave factor >0.04 or <−0.04. All samples that failed QC after the wave adjustment procedure were removed. We excluded CNV calls when they failed stringent quality control (QC) criteria: <5 probes, <100 kb size. To ensure that we were working with high-confidence CNVs, we excluded any CNV for which the difference of the log likelihood of the most likely copy number state and the less likely copy number state was less than 10 (generated using the – conf function in PennCNV), as these CNVs were likely to be unreliable at the current array resolution. Due to the complications of hemizygosity in men and X-chromosome inactivation in women, all analyses were restricted to autosomes. We excluded any CNV loci that overlapped with any of the following regions by ≥50% of its length (Need et al. 2009) if: (1) they were within centromere proximal bands or overlapping immunoglobulin regions that are both known to be prone to false positives due to somatic mutations; (2) some centromeric and telomeric regions that are not well mapped; and (3) genomic regions coding for immunoglobulin genes have previously been shown to be potential sites of false-positive PennCNV calls (Wang et al. 2007).

We removed three control samples that had an excessive number of CNVs (CNV >26). The number was considered excessive if the samples had a number of CNV calls exceeding the mean by three standard deviations. The resulting cutoff for the number of CNVs was 1603 for PennCNV. All CNVs >1 Mb were inspected manually. Samples with excessive aggregate length of CNVs, as well as samples with CNVs >7.5 Mb (which likely correspond to karyotyping abnormalities) were dropped from the analysis. The steps in the quality control process are summarized in Table S1.

For the purpose of rare burden analysis, CNVs with more than 50% of their length overlapping Segmental Duplications (Human reference NCBI36/hg18; UCSC genome browser) were discarded as previously described, and CNVs found in more than 1% of cases and controls were not considered further. Consequently, we removed 617 CNV from the total 1603 qualified CNV. Finally, we studied 986 CNV defined as rare CNV using a total of 261 cases (176 men and 85 women) and 171 controls (72 men and 99 women) that passed all QC steps. The female/male ratio and demographic and clinical characteristics (e.g., age at onset) in the dataset that passed all QC steps remained similar to original dataset (Table 1).

**Table 1:** Demographic and clinical characteristics of study participants

	Original sample		Samples that passed CNV QC	
		
	Case	Control	Case	Control
Subjects (%)	268 (60.1)	178 (39.9)	261 (60.4)	171 (39.6)
Mean age at onset/examination, Year ± SD^1^	59.9 ± 12.1	69.8 ± 8.8	60.0 ± 12.2	69.9 ± 8.9
Male (%)	179 (66.8)	76 (42.7)	176 (67.4)	72 (42.1)
Family history (%)	53 (19.8)	13 (7.3)	53 (20.3)	13 (7.6)
Proportion of EOPD^2^	0.245		0.244	
Genotyping rate	99.85			
Total sample	446		432	

1 Age at onset for cases and age at examination for controls.

2 EOPD: early at onset of PD cases, with age at onset younger than or equal to 50.

### Global CNV burden analysis

To determine whether or not cases show a greater genome-wide burden of CNVs compared to controls, CNV burden analyses were conducted using PLINK v1.07 (Purcell et al. 2007) and statistical significance was assessed using a permutation procedure (one-sided, 100,000 permutations). *P*-values were estimated for the number of CNVs per individual (CNV rate), CNV sample proportion (fraction of samples with one or more CNVs) and for the total or average size ranges of CNV calls. We further explored subsets of rare CNVs in two additional frequency ranges: 2–6 occurrences and single occurrences. Single occurrences were defined as CNVs that did not overlap any other CNVs in the dataset for more than 50% of their length. We also assessed duplications and deletions separately in the same frequency ranges. For these specific sets, deletions were considered as single occurrences, even if they overlapped with a duplication for more than 50%; likewise, duplications were considered as single occurrences even if they overlapped with a deletion for more than 50%. Therefore, the number of single-occurrence deletions and duplications do not sum to the number of single-occurrence CNVs in the frequency filtered sets that consider both CNV types. The set with 2–6 occurrences was obtained by selecting all rare CNVs that overlapped 6 or fewer other CNVs by at least 50% of their length, and subsequently removing CNVs that met the definition of single occurrence. Gene count evaluated the average number of genes intersected by CNVs per case compared to that for control sample. Genic regions were identified based on RefSeq annotations (UCSC, v. April 2009, NCBI v36, hg18) and were defined by the outermost boundaries of the full set of transcript isoforms. Gene boundaries were extended with a 20 kb flanking region on either side. Case–control comparisons were assessed using a permutation procedure for statistical significance of one-sided tests (i.e., hypothesizing that cases will show greater burden of rare CNVs than controls). For each of 100,000 permutations, samples were randomly reassigned as either case or control. In a similar manner, we also analyzed subsets of rare CNVs, deletions-only, and duplications-only, separately. For rare CNVs, we examined whether CNV frequencies were elevated in PD cases against AJ controls as well as control subjects in the Database of Genomic Variants (DGV) where control subjects of diverse populations are included. While we recognize that these randomly selected controls in the DGV were not screened for the presence of PD, the number of PD patients in DGV will be low. A total of 986 rare stringent CNVs in a total sample set of 432 (261 cases and 171 controls) were used in the analyses.

### Ingenuity pathway analysis

Ingenuity pathway analysis (IPA) software (http://www.ingenuity.com) was used to search for biological relationships among genes with CNV identified in PD cases as a single occurrence or ≤5 PD cases and no controls. A gene list (Table S2) was entered into a “My Pathway” analysis in IPA. Restricting species to human and allowing for findings among chemicals, the Path Explorer tool under the Build tab was used to search among the Ingenuity knowledge base and external databases to identify the shortest pathways among the genes with either no or 1 intervening molecule. Links between genes represent protein–protein interactions or indicate that 1 gene influences phosphorylation of the connected gene.

### CNV validation

CNVS were only validated in the original case or control identified with a specific CNV (by Penn CNV). Since the majority of CNVs identified, with the exception of OVOS2, were rare (and singleton) it was not possible to validate all CNVs and we selected a subset of nine CNV regions (CNVRs) (listed in Table 5) identified from the rare CNVR burden analysis to validate experimentally.

We validated a total of nine CNVRs identified by PennCNV, in the initial genome-wide screen (Figs. S3A and B and 1C). We used TaqMan® Copy Number Assays from ABI (Applied Biosystems Inc., Carlsbad, CA) on the ABI Prism® 7900HT Sequence Detection System for validation in cases and controls. A VIC-labeled Copy Number Assay for RNase *P* was selected to be an endogenous control as it performed in the same reaction with gene-specific assays. FAM-labelled Probe(s) were selected for each CNVR and are shown in Table S3. Each sample was assayed with four replicates. The CNV calls were generated with SDS software and CopyCaller™ from ABI (Applied Biosystems Inc., Carlsbad, CA). A control sample was used as a reference for a copy number of 2. This control sample was already examined by SNP microarray to contain two copies of candidate CNVRs. In order to make CNV calls in CopyCaller™ software, a confidence score of greater than 0.95 was required with four replicates.

**Figure 1:**
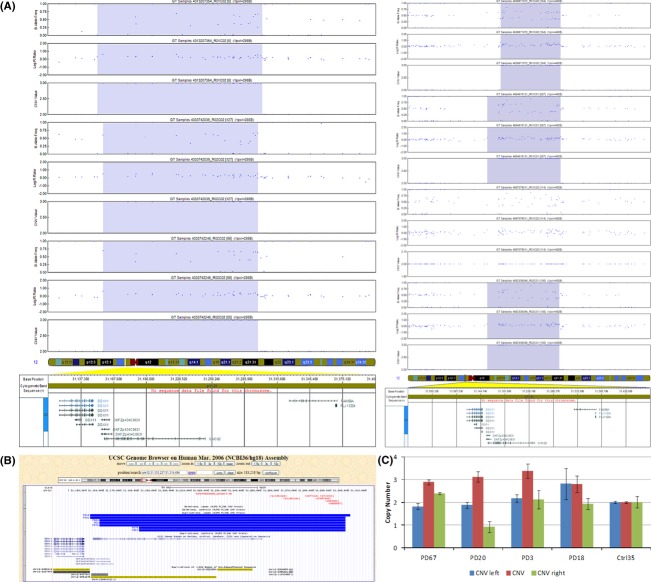
CNVs at *OVOS2* in PD patients detected in PD patients. (A) Illumina Genome studio cnvPartition CNV Analysis Plug-in software was used to visualize CNVs. Duplications are indicated by B allele frequency, LogR ratio and CNV value for each PD patient. (B) Schematic of *OVOS2* contained in the CNV region. Screenshot of the UCSC genome browser based on NCBI36/hg18 assembly (Chr12: 31,101,458–31,307,623). (C) Validation of the CNVs at *OVOS2* identified. A heterozygous duplication (copy number = 3) in four PD cases (PD67, PD20, PD3, and PD18) is shown in addition to a normal control (ctrl35; copy number = 2). Three PD cases (PD31, PD72, and PD74) did not have sufficient genomic DNA (from blood) to perform validation.

## Results

### Demographic and clinical characteristics of cases and controls included in the CNV analysis

Demographic and clinical characteristics of subjects were comparable between the initial dataset and the dataset that passed QC procedures. A total of 432 of 446 passed QC procedures and were included in the CNV analysis (*n* = 432; 261 cases and 171 controls) (Table 1). Overall, controls were older than cases and were more likely to be female. Of the PD cases included in the study, 24.5% were early onset PD cases (AAO ≤50 years). PD cases were twice as likely as controls to have a family history of PD in a first-degree relative.

### Characteristics and distribution of CNVs identified in the AJ case–control population

Table 2 summarizes the characteristics of CNVs identified in the sample. Overall, a total of 1603 CNVs (length ≥100 kb) were identified in the final sample. The average number of CNVs and the average CNV size did not differ significantly between cases and controls (Table 2). Overall, cases had more CNVs (*n* = 988; 61.6%), including deletions and duplications, than controls (CNV: *n* = 615; 38.4%) but the average number of CNVs per individual did not differ between cases and controls (Table 2). The CNV span size was significantly larger in cases compared to controls (Table 2).

**Table 2:** Characteristics of all CNVs with CNV size ≥100 kb

	Cases			Controls				
				
CNV	Number of CNVs (% total CNV)	Cases with CNV	Mean number CNV/case	Number of CNVs (% total CNV)	Controls with CNV	Mean number CNV/control	Total CNVs	Fisher's *P*
Deletion (%)	446 (27.8%)	193	2.3	248 (15.5%)	125	2	694	0.9112
Homozygous deletion	52	51	1	24	23	1	76	0.1173
Heterozygous deletion	394	173	2.3	224	118	1.9	618	0.6004
Duplication (%)	542 (33.8%)	209	2.6	367 (22.9%)	140	2.6	909	0.7085
Heterozygous duplication	534	208	2.6	362	140	2.6	896	0.6202
Homozygous duplication	8	6	1.3	5	4	1.3	13	1.0000
Total CNV	988 (61.6%)	248	4	615 (38.4%)	158	3.9	1603	0.3029
CNV span number SNP	5–449			5–274			5–449	
Mean number SNP/CNV	41.9			40.2			41.3	
CNV span size (kb)	100.1–1747.0			100.3–1070.4			100.1–1747.0	
Mean CNV size (kb)	180.1			186.7			182.7	
CNV median size (kb)	140.7			145.3			141.6	
CNV frequency (100–250 kb)	85.83			83.41			84.9	
CNV frequency (250–500 kb)	12.65			14.15			13.23	
CNV frequency (500–1000 kb)	1.01			2.11			1.43	
CNV frequency (>1000 kb)	0.51			0.33			0.44	

Fisher's *P*: *P*-value compares the number of CNVs in the cases to the controls.

### Characteristics and distribution of rare CNVs in the AJ case–control population

Stringent CNVs that passed all QC filters (≥5 probes, ≥100 kb size) were considered rare if they occurred at a frequency of ≤1% in the dataset (*n* = 432 subjects) and did not overlap, with ≥50% of its length with segmental duplications (human reference NCBI36/hg18; UCSC genome browser). Overall, 986 CNVs in a total of 432 subjects met criteria for rare CNV after sample and CNV quality filtering. Table 3 gives an overview of the characteristics of rare CNVs in PD cases and controls. The average number of CNVs per individual was 3 and size was 205 kb. A total of 600 CNVs were present in 203 cases (average CNV size 204.6 kb, median CNV size 153.2 kb) and 386 CNVs in 139 controls (average CNV size 205.7 kb, median CNV size 158.1 kb). The average number of CNVs per individual did not differ in cases compared to controls. Overall, CNVs were larger in cases as measured by CNV span size (span CNV length: 100.1–1747.0 kb) than in controls (span CNV length: 100.3–1070.4 kb).

**Table 3:** Characteristics of rare CNV (≤1% in the dataset of 432 subjects) with length ≥100 kb

	Cases			Controls				
				
CNV	Number of CNVs (% total CNV)	Cases with CNVs	Mean number CNV/case	Number of CNVs (% total CNV)	Controls with CNVs	Mean number CNV/control	Total CNVs	Fisher's *P*
Deletion (%)	245 (24.8%)	115	2.1	143 (14.5%)	85	1.7	388	0.2781
Homozygous deletion	1	1	1	2	2	1	3	0.5652
Heterozygous deletion	244	115	2.1	141	84	1.7	385	0.3244
Duplication (%)	355 (36.0%)	164	2.2	243 (24.6%)	109	2.2	598	0.9188
Heterozygous duplication	348	162	2.1	239	109	2.2	587	0.7607
Homozygous duplication	7	5	1.4	4	3	1.3	11	1.0000
Total CNV	600 (60.9%)	203	3	386 (39.1%)	139	2.8	986	0.3990
CNV span number SNP	5–449			5–274			5–449	
Mean number SNP/CNV	49.1			45.8			47.8	
CNV span size (kb)	100.1–1747.0			100.3–1070.4			100.1–1747.0	
Mean CNV size (kb)	204.6			205.7			205	
CNV median size (kb)	153.2			158.1			155.6	
CNV frequency (100–250 kb)	78			76.42			77.38	
CNV frequency (250–500 kb)	19.5			19.95			19.68	
CNV frequency (500–1000 kb)	1.67			3.11			2.23	
CNV frequency (>1000 kb)	0.83			0.52			0.71	

Fisher's *P*: *P*-value compares the number of CNVs in the cases to the controls.

### Global rare CNV burden analysis

We assessed the impact of rare CNV in cases compared to controls using three primary measures of global CNV burden: the number of CNVs per individual, the estimated CNV size, and the number of genes affected by CNVs (Table 4, Table S4). No significant differences were found in the gene counts affected by CNVs (Table S4). However, we observed a significant increase in total CNV size for deletions in cases compared to controls (1.37-fold increase, empirical *P* = 0.019).

**Table 4:** Global rare CNV burden analysis

					CNV rate^1^			CNV sample proportion^2^			Total CNV Size (kb)			Average CNV size (kb)		
								
Type	Classification	Case CNV	Control CNV	Total CNV (*n*)	Empirical *P*	Case/control ratio	Baseline rate (control)	Empirical *P*	Case/control ratio	Baseline rate (control)	Empirical *P*	Case/control ratio	Baseline rate (control)	Empirical *P*	Case/control ratio	Baseline rate (control)
All	All	600	386	986	0.451	1.02	2.26	0.839	0.96	0.81	0.313	1.06	571.2	0.779	0.96	207.2
Deletions	All	245	143	388	0.279	1.12	0.84	0.895	0.89	0.50	**0.019**	**1.37**	295.6	0.341	1.03	182.4
Duplications	All	355	243	598	0.631	0.96	1.42	0.644	0.98	0.64	0.675	0.94	497.9	0.958	0.89	228.3
CNV frequency^3^																
All	1×^3^	114	61	175	0.299	1.22	0.36	0.451	1.05	0.20	0.454	1.04	435.2	0.818	0.83	300.3
	2–6×^3^	470	322	792	0.675	0.96	1.88	0.896	0.94	0.78	0.432	1.02	473.8	0.380	1.01	189.7
Deletions only	1×	62	32	94	0.285	1.27	0.19	0.218	1.28	0.11	0.451	1.04	317.6	0.537	1.02	214.8
	2–6×	175	108	283	0.375	1.06	0.63	0.946	0.84	0.44	**0.005**	**1.38**	243.3	0.143	1.08	173.5
Duplications only	1×	70	35	105	0.332	1.31	0.20	0.673	0.93	0.14	0.351	1.15	440.0	0.774	0.84	335.4
	2–6×	279	208	487	0.830	0.88	1.22	0.849	0.93	0.62	0.724	0.92	412.3	0.818	0.94	206.2
CNV size																
All	100–500 kb	585	372	957	0.416	1.03	2.18	0.802	0.96	0.81	0.284	1.07	502.9	0.558	0.99	185.6
	≥500 kb	15	14	29	0.864	0.70	0.08	0.872	0.71	0.08	0.076	1.26	768.7	**0.046**	**1.24**	723.1
	≥1 Mb	5	2	7	0.426	1.64	0.01	0.426	1.64	0.01	0.157	1.21	1039.0	0.157	1.21	1039.0
Deletions only	100–500 kb	241	142	383	0.297	1.11	0.83	0.871	0.90	0.49	0.055	1.27	289.3	0.443	1.01	174.7
	≥500 kb	4	1	5	0.342	2.62	0.01	0.342	2.62	0.01	0.518	1.22	830.9	0.518	1.22	830.9
	≥1 Mb	1	0	1	0.602	N/A	0.00	0.602	N/A	0.00	0.602	N/A	0.0	0.602	N/A	0.0
Duplications only	100–500 kb	344	230	574	0.568	0.98	1.35	0.548	1.00	0.63	0.589	0.97	421.5	0.843	0.95	198.7
	≥500 kb	11	13	24	0.941	0.55	0.08	0.954	0.55	0.07	0.106	1.25	763.6	0.076	1.19	714.2
	≥1 Mb	4	2	6	0.555	1.31	0.01	0.555	1.31	0.01	0.159	1.09	1039.0	0.159	1.09	1039.0

Samples and CNVs that failed stringent quality criteria had previously been excluded, maintaining CNVs with ≥100 kb in size. We tested for global CNV burden in AJ cases (*n* = 261) compared to controls (*n* = 171) considering rare CNVs, that is, CNVs that are present in <1% of our total sample. Analyses were further stratified according to CNV type (deletions-only and duplications-only) and frequency (CNV observed 2–6 times and in isolated cases). Genome-wide *P*-values were estimated by permutation (one-sided, 100,000 permutations), and report on four tests for CNV burden: number of CNVs (CNV rate), CNV sample proportion (proportion of samples with one or more CNVs), total kb size spanned, and average CNV size. The baseline rate in controls and the fold increase in cases (case/control ratio) are listed for each analysis.

1 #CNVs per sample.

2 Proportion of samples with one or more CNVs.

3 CNV frequency: CNV observed 2–6 times in the total sample (2–6×) and one time (1×) Significant differences (*P* < 0.05) are indicated in bold.

In the subgroup analysis based on the CNV frequency, 175 CNVs (94 deletions and 105 duplications) were observed once in this dataset, whereas 792 CNVs (283 deletions and 487 duplications) were observed in two to six samples either in cases or controls. CNVs with a frequency ≥2 (2–6×) were significantly larger in cases than controls, indicating that some could be pathogenic or reflect underlying biology (1.38-fold increase, empirical *P* = 0.005). The average size for CNVs ≥500 kb in length was also significant in cases compared to controls (1.24-fold increase, *P* = 0.046). We examined the clinical and demographic characteristics of PD cases and controls with CNV ≥500 kb in addition to regions and genes disrupted by the CNV (Table S5). A total of four cases and two controls carried overlapping CNVs at Chr15: 18607432-19872353 (Table S5). CNVs in the 4 cases (784 kb, 918 kb, ∼1 Mb and 1.2 Mb, respectively) were significantly larger and encompassed more genes compared to CNVs in two controls (506 kb and 657 kb, respectively). Two subjects, one case (AAO = 62 years) and one control (age at evaluation 56 years), carried duplications (CN = 3, heterozygous duplication) of the entire *CNTN6* gene. A total of two cases and three controls carried duplications of the Zinc finger protein 479 pseudogene (*LOC643955*). Given the similar frequency in cases and controls and annotation as a pseudogene this CNV is unlikely to represent a pathogenic event. All other CNVs >500 kb were unique events and showed no overlap with CNVs in other subjects.

**Table 5:** Global rare CNV region burden analysis

Gene	Position	Number case with CNV	Number case without CNV	Number control with CNV	Number control without CNV	Fisher test	*P*-value^1^	*P*-value^2^
*CFH*	chr1:194887630-194983257	3	258	0	171	0.2195	1	1
*BCHE*	chr3:166973385-167037947	4	257	0	171	0.1320	0.1334	0.9437
*SDK1*	chr7:3307605-4275157	3	258	0	171	0.2195	0.2184	0.9993
*DLC1*	chr8:12985242-13416766	3	258	0	171	0.2195	0.2187	0.9993
*SGCZ*	chr8:13991743-15140163	3	258	0	171	0.2195	0.2187	0.3004
*CYP2E1*	chr10:135190856-135202610	3	258	0	171	0.2195	0.2195	0.9993
*SYCE1*	chr10:135217394-135232866	3	258	0	171	0.2195	0.2195	0.9993
*PPYR1*	chr10:46503539-46508326	5	256	0	171	0.0793	0.0793	0.8020
*ANXA8*	chr10:46577989-46594128	3	258	0	171	0.2195	0.1310	0.9437
*ANXA8L1*	chr10:46577994-46594046	3	258	0	171	0.2195	0.1310	0.9437
*OVOS2*	chr12:31158726-31250355	7	254	0	171	0.0284	0.0288	0.4952

1 Empirical *P*-value, per region.

2 Empirical *P*-value, corrected for all tests.

### Rare CNVR burden analysis

We also tested for increased global burden of rare CNVRs. Here, 351 nonredundant CNVRs were constructed by merging overlapping rare CNVs present in the total sample of cases and controls (*n* = 432 samples). Statistical significance for each gene or CNV was assessed by Fisher's exact test (Table 5). From these analyses, ovostatin 2 (*OVOS2*) was identified as a significant risk factor for PD (*P* = 0.028). Seven PD cases with no reported family history in a first degree relative carried overlapping duplications (copy number = 3) (heterozygous duplication) at *OVOS2*; CNV length range: 118–148 kb) at *OVOS2* (Chr12p11.21) (Table 6, Fig. 1). CNVs at *OVOS2* were not observed in 171 controls. We compared the demographic and clinical characteristics of the seven PD cases carrying the *OVOS2* CNV to PD cases without the *OVOS2* CNV (Table S6). Overall, the clinical and demographic characteristics were similar except that all *OVOS2* CNV carriers were male (100%) and none had a family history of PD in a first-degree relative. A total of seven SNPs in the region reached significance with *P* < 0.05 (data not shown; top SNP, rs713334, *P* = 1.85 × 10^−3^, OR = 1.68, 95% CI = 1.21–2.34). SNPs in the surrounding genes, *DDX11* and *FAM60A*, were not significant. We confirmed that the CNV at *OVOS2* identified in the seven sporadic PD cases represents a recurrent CNV and not a founder event by examining haplotypes in the region (Table S7). In the database of genomic variants (DGV), CNVs in the *OVOS2* region are rare, with a frequency ranging from 0.00057–0.10, and a mean of 0.0028 (208 CNVs/73458 controls), in five studies with large control datasets (sample size >400). Our sample size is comparable to one of the DGV datasets (Jakobsson et al. 2008) which included 485 control subjects from the Human Genome Diversity panel (HGDP) (Li et al. 2008) and which reported a frequency of 0.0034 for *OVOS2* CNVs. Although there is no NCBI RefSeq NM or XM model for *OVOS2*, Homo sapiens cDNA sequences in GenBank, coaligned on the genome and clustered in a minimal nonredundant way support at least 14 alternative spliced variants. The sequence of the *OVOS2* gene is defined by 106 GenBank accessions from 94 cDNA clones, from testis (seen 24 times), lung (22), carcinoid (18), eye (11), pooled germ cell tumors (7), retina (5), brain (3), and 22 other tissues. The *OVOS2* gene also shows a high degree of conservation across mammalian species providing further evidence that *OVOS2* is a functional gene.

**Table 6:** Clinical characteristics of PD cases with CNV at *OVOS2*.

IID	Family history	Gender	Status	Age at onset	Number SNP	CNV length (bp)	CN	Conf.	Gene	CNV region
PD31	No	M	Case	54	54	145,949	3	52.8	*OVOS2*	chr12:31152226-31298174
PD18	No	M	Case	NA	33	148,621	3	84.3	*OVOS2*	chr12:31152226-31300846
PD3	No	M	Case	45	31	140,621	3	95.9	*OVOS2*	chr12:31157554-31298174
PD20	No	M	Case	63	31	140,621	3	77.4	*OVOS2*	chr12:31157554-31298174
PD72	No	M	Case	46	53	140,621	3	177.6	*OVOS2*	chr12:31157554-31298174
PD67	No	M	Case	56	52	118,024	3	82.6	*OVOS2*	chr12:31180151-31298174
PD74	No	M	Case	73	52	118,024	3	120.2	*OVOS2*	chr12:31180151-31298174

CN, copy number; Conf., confidence score.

### CNVs in known PD genes

We did not identify novel or previously reported CNVs in the Mendelian PD genes, *SNCA*, *PARK2*, *DJ-1*, and *TH1* in AJ PD cases in this study. In a CNV study of PD from Pankratz et al. (2011) single CNVs at the PARK2 locus were identified in 10 of the 396 independent cases and 8 of the 856 controls, yielding an odds ratio of 2.7 (*P* = 0.03). In this study, we have power to detect CNVs at PARK2 with a RR of ≥1.6 assuming a prevalence of 0.25 (Table S8). CNVs were also absent from all other Mendelian PD genes including PTEN-induced putative kinase (*PINK1*; *PARK6*) (Valente et al. 2004), leucine rich repeat kinase 2 (*LRRK2*; *PARK8*) (Paisan-Ruiz et al. 2004), and glucocerebrosidase (*GBA*) (Clark et al. 2007). With the exception of the *MAPT* region at Chr17q21.3 we did not identify CNVs in candidate and risk gene associations identified by GWAS (Lill et al. 2012). In the *MAPT* region at Chr17q21.3, we identified one PD case sample with a large deletion spanning *NSF* and *WNT3*. Previously, we identified this region (*NSF* and *WNT3*) as a “top hit” in a GWAS study (*NSF*, rs183211, *P* = 7.2 × 10^−5^, OR = 0.54, 95% CI = 0.40–0.73) (Liu et al. 2011).

Contribution of copy number variants identified in PD cases (but not in controls) at two gene regions reported by Pankratz et al. (2011) also overlapped with CNVs identified in PD cases in this study: (1) a CNV in the *FPR3* gene (chr19: 56972447-56988627; length 16,180 bp in two PD cases) reported in the Pankratz study overlaps with a CNV (CN = 3; 332,470 bp; chr19: 56972447-57304917) that we identified in one AJ PD Case: PD 28, male patient, 62 years old, and (2) a CNV in the *MACROD2* gene identified in three PD cases reported in the Pankratz study, chr20:14662457-14706111 (length 43,654 bp) was found to overlap with a CNV (CN = 3; 223, 348 bp; chr20:14679595-14902943) in one AJ PD case: PD54, female patient, and 72 years old.

### Candidate rare genic CNVs identified in PD cases

We identified all rare single occurrence genic CNVs and “recurrent” CNVs in ≤5 PD cases that were absent in controls (Table S9). Overall, 81 PD cases (39.9% = 81/203) carried a rare genic CNV that was absent in controls. A total of 28 PD cases (34.6% = 28/81) had multiple CNV events. The majority of PD cases with a rare genic CNV (*n* = 67; 82.7% = 67/81) reported no family history in first degree relative. Overall, only 14 of 81 PD cases (17.3% = 14/81) had a family history of PD in a first-degree relative. Family members were not available to examine inheritance or demonstrate that these CNVs are de novo occurrences in sporadic cases. We also examined controls for rare genic CNVs that were absent in cases (Table S9). A total of 33 controls (23.7% = 33/139) carried a rare genic CNV that was absent in cases. A total of eight controls (24.2% = 8/33) had multiple CNV events.

### Functional and disease pathway analysis

**Figure 2:**
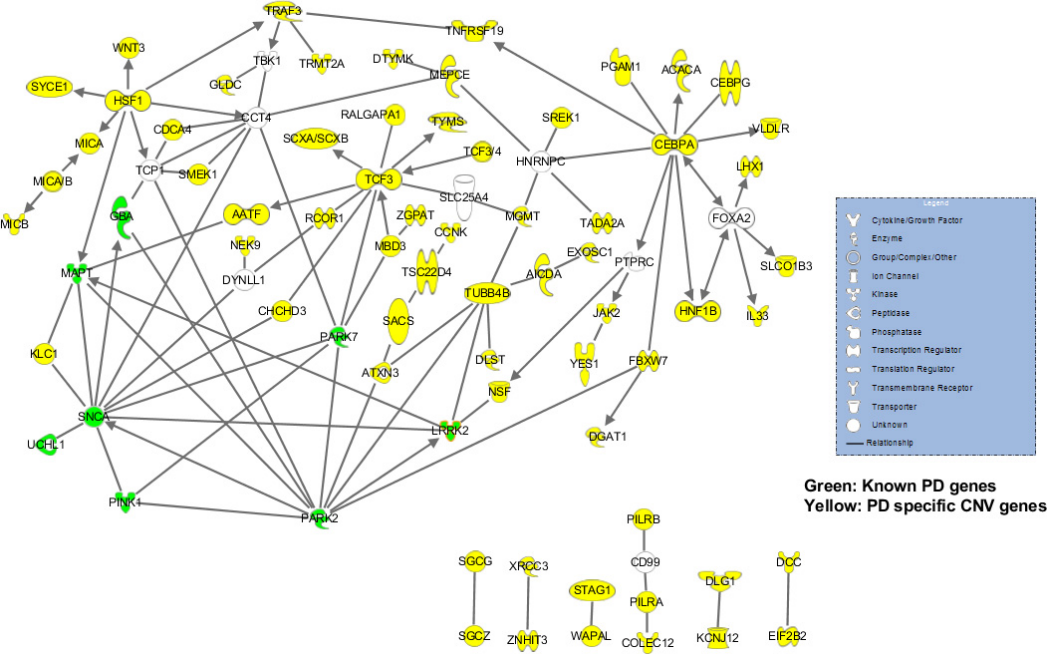
Ingenuity pathway analysis. Interaction of all genes included in all PD specific CNVs with known PD genes.

We further examined the candidate rare genic CNVs identified in PD cases to determine functional relevance and whether they are predicted to disrupt genes that are in disease pathways relevant to PD. A total of 338 genes with CNVs were included in the ingenuity pathway analysis (Table S2). We ranked functional and disease categories based on enrichment with the candidate genes (Table S10). The top 10 ranked categories included functional and disease categories related to glucose metabolism, diabetes, and cell death including the following: glucose metabolism disorder, diabetes mellitus, disorder of pancreas, cell death, and non-insulin diabetes mellitus. IPA was also used to search for biological relationships among the candidate rare genic CNVs with previously identified Mendelian PD genes (*SNCA*, *PARK2*, *LRRK2*, *UCHL1*, *PINK1*, *DJ1*, *MAPT*, and *GBA*) (Fig. 2). Paths between genes represent protein–protein interactions or phosphorylation. This network suggests that *ATXN3* and *FBXW7* may be part of the same disease pathway as *PARK2* (Parkin), *CHCHD3* and *KLC1*as *SNCA*, *AATF*, *HSF1*, and *KLC1* as *MAPT*, *TCF3*, and *MBD3* as *DJ-1* (*PARK7*), *TUBB4B* and *NSF* as *LRRK2* while other genes (*SGCZ*, *BCHE*, *DLC1*, *CYP2E1*, *NRXN1*, *SGCG*, and *JAK2*) that we identified may influence risk of PD via another mechanism. Overall, nine (11.1%) of 81 PD cases carried a rare genic CNV that mapped to the same network as known PD genes. We examined differential expression of the candidate genic CNVs that we identified using published PD microarray datasets and the National Center for Adult Stem Cell Research Parkinson's Review database (http://ncascr.griffith.edu.au/pdreview/2009/) (Sutherland et al. 2009). Genes showing direct interaction including *ATXN3*, *FBXW7*, *HSF1*, *KLC1*, and *MBD3* showed significant differential expression in one or more datasets (Table S11). Other candidate genic CNVs that we identified including *NRXN1*, *BCHE*, *CYP2E1*, *NSF*, and *JAK2* also showed significant differential expression suggesting that these genes are in pathways that are altered in PD pathogenesis (Table S11).

## Discussion

To evaluate the contribution of CNVs to the risk of PD in the AJ population, we analyzed an AJ dataset from NY comprised of 446 samples (268 PD cases and 178 controls) with genotypes for >600,000 SNPs across the genome. We hypothesized that “founder” CNVs or risk CNVs shared by multiple AJ cases would be identified in this population. However, our study suggests that there is a significantly increased rate of de novo sporadic and recurrent rare genic CNVs in AJ PD cases compared to controls. One study that assessed CNV in Schizophrenia in an AJ population also observed mostly de novo events and no evidence for “common” or “founder” CNV risk variants (Mulle et al. 2010). Limitations of this study are that we were not able to test transmission of CNVs in other family members to confirm inheritance in familial cases or de novo occurrence in sporadic cases.

To date only one other published study has performed a genome wide evaluation of CNVs in PD. In the published study from Pankratz et al. (2011), in a genetically heterogeneous familial PD population from North America, CNVs at *PARK2*, were identified as a susceptibility locus. This study did not replicate the *PARK2* locus as a PD susceptibility gene, which may reflect differences in study design (e.g., family history), ethnicity (Jewish vs. non-Jewish) or statistical power. With the exception of the *MAPT* region at 17q21.3 we did not identify novel or previously reported CNVs in known Mendelian or GWAS PD loci. In the *MAPT* region at Chr17q21.3 we identified one case sample with a large deletion spanning *NSF* and *WNT3*. Previously, we identified this region (*NSF* and *WNT3*) as a “top hit” in a GWAS study (Liu et al. 2011).

For rare CNVRs, global burden was elevated and an association between PD and *OVOS2* was observed (Chr12p11.21) (empirical *P* = 0.028). However, the association between PD and *OVOS2* was not significant after multiple testing. Functionally *OVOS2* is candidate for PD because it belongs to the inhibitor family I39 (alpha_2_-macroglobulin [A2M] family) of endopeptidase binding molecules (Rawlings et al. 2012). In mammals, A2M functions as a broad spectrum endopeptidase-binding molecule that mediates the clearance of endopeptidases from the plasma (Rawlings et al. 2012). A2M is a component of Lewy bodies, a hallmark of the neuropathology of PD and is considered a candidate gene for Alzheimer disease; however, the association between SNPs in *A2M* and risk for PD or AD remains unknown with positive and negative reports of association. Given the unknown significance of this finding it will be important to replicate this result in different PD populations.

Ingenuity pathway analysis showed that the rare genic CNVs identified in this study mapped to the same pathways as previously identified PD genes (*SNCA*, *PARK2*, *LRRK2*, *UCHL1*, *PINK1*, *DJ1*, *MAPT*, and *GBA*). The IPA network identified *ATXN3* and *FBXW7* in the same disease pathway as *PARK2* (Parkin); *CHCHD3* and *KLC1* as *SNCA*; *AATF*, *HSF1*, and *KLC1* as *MAPT*; *TCF3* and *MBD3* as *DJ-1* (*PARK7*); *TUBB4B* and *NSF* as *LRRK2*. We also examined differential expression of the candidate genic CNVs that we identified using published PD microarray datasets and the National Center for Adult Stem Cell Research Parkinson's Review database (http://ncascr.griffith.edu.au/pdreview/2009/) (Sutherland et al. 2009). Many of the candidate genes that we identified showed differential expression in more than one PD microarray dataset. Of the genes that did not map to the Mendelian PD gene IPA network many are putative candidates based on either function or role in other neuropsychiatric or neurodegenerative disease. For example, in one PD case (AAO 75 years), we observed a 239 kb deletion (CN = 1) on chr2p16.3 (50753861-50993732) (Table S10) encompassing the distal region of the neurexin1 gene (*NRXN1*). *NRXN1* encodes a neuronal cell surface protein involved in cell recognition and cell adhesion and genome-wide CNV studies have previously implicated *NRXN1* deletions in autism and schizophrenia (Ching et al. 2010; Magri et al. 2010) and in AD (Ghani et al. 2012). In three PD cases, we identified a duplication encompassing *CYP2E1*. Cytochrome P450 2E1 (*CYP2E1*), which is located in dopamine containing neurons in the substantia nigra, has been hypothesized to be of importance for the pathophysiology of PD, either by its production of reactive oxygen species (ROS) or by its capability to detoxify putative neurotoxins.

In summary, this study suggests that de novo and recurrent rare genic CNVs may contribute to PD susceptibility. Since there are few published studies that have evaluated the contribution of rare CNVs to PD in different populations follow-up and replication studies are of great importance.

## Supporting Information

**Figure S1.** Principal component analysis in the entire sample (*n* = 446).

**Figure S2.** Principal component analysis delineates AJ GWAS samples and overlap with HapMap samples.

**Figure S3.** Summary of CNVs validated by qPCR. (A) Validation of CNV regions identified in the global rare CNV region burden analysis (Table 5). (B) Validation of the SGCZ CNV region in 3 PD cases (PD56, PD71, and PD77) and 1 control.

**Table S1.** Steps in the CNV QC process.

**Table S2.** Candidate genes included in the IPA analysis.

**Table S3.** List of primers and probes used for qPCR validation of CNVs. (A) Taqman assays. (B) Custom assays. (C) Roche universal probe library probe and primers for *SGCZ* assay.

**Table S4.** Global rare CNV burden analysis and gene counts.

**Table S5.** CNV ≥500 kb identified in cases and controls.

**Table S6.** Demographic and clinical characteristics of PD *OVOS2* CNV carriers compared to PD noncarriers.

**Table S7.** *OVOS2* haplotype analysis.

**Table S8.** Power analysis to detect CNVs at PARK2.

**Table S9.** Rare single occurrence CNVs or CNVs identified in ≤5 PD cases that are absent in controls.

**Table S10.** IPA functional and disease categories enriched with PD candidate genes.

**Table S11.** PD microarray datasets for candidate genes using the database from http://ncascr.griffith.edu.au/pdreview/2009/.
