# Supplementary figures and images for: Increased rate of sporadic and recurrent rare genic copy number variants in Parkinson's disease among Ashkenazi Jews
Source: Mol Genet Genomic Med. 2013 Jun 7;1(3):142–54. doi: 10.1002/mgg3.18 (PMC3782064; doi:10.1002/mgg3.18)

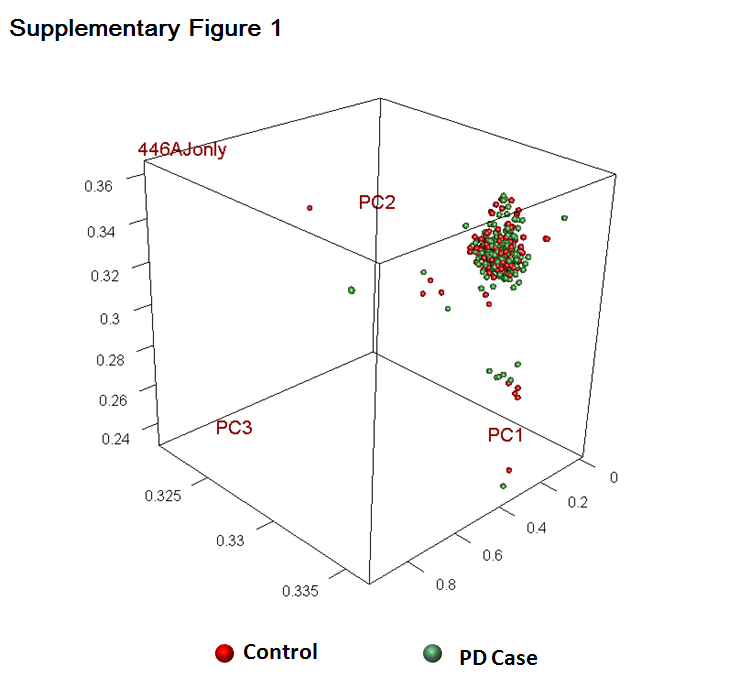

Supplement: Supplementary file 1 [file mgg30001-0142-SD1.tif]

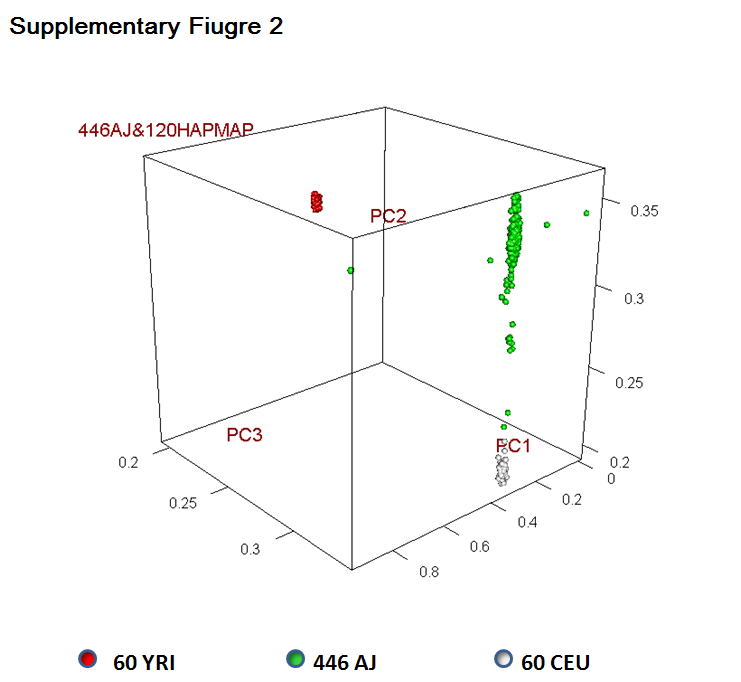

Supplement: Supplementary file 2 [file mgg30001-0142-SD2.tif]

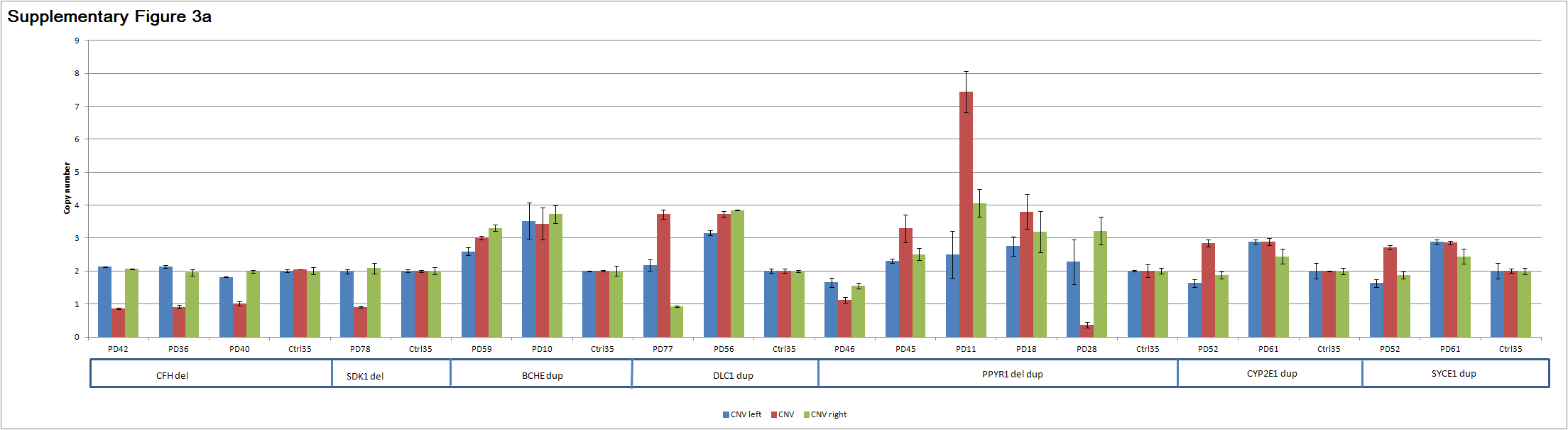

Supplement: Supplementary file 3 [file mgg30001-0142-SD3.tif]

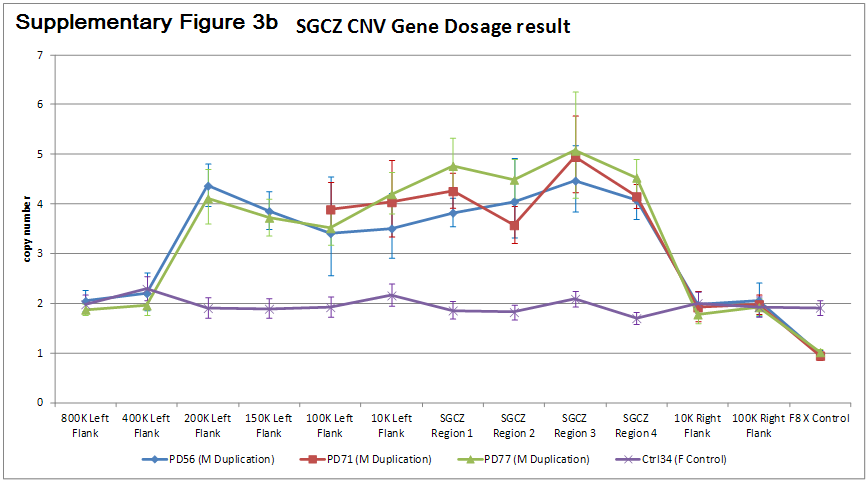

Supplement: Supplementary file 4 [file mgg30001-0142-SD4.tif]
