# Supplementary material for: Increased rate of sporadic and recurrent rare genic copy number variants in Parkinson's disease among Ashkenazi Jews
Source: Mol Genet Genomic Med. 2013 Jun 7;1(3):142–54. doi: 10.1002/mgg3.18 (PMC3782064; doi:10.1002/mgg3.18)
Supplement: Supplementary file 5 [file mgg30001-0142-SD5.docx]

Power Calculations to detect CNVs at the PARK2 locus for the AJ case control sample (268 cases and 178 controls) for a range of MAF (0.05-0.10), relative risk (RR)(1.2-2.1) and significance levels assuming a prevalence of 0.25.

| **268 cases vs 178 controls power** | | | |  |  |  |  |  |  |  |
| --- | --- | --- | --- | --- | --- | --- | --- | --- | --- | --- |
| 178/268 | = | 0.664179104 |  | ***maf=0.05*** |  |  |  |  |  |  |
| Prevalence | 0.3 | 0.3 | 0.3 | 0.3 | 0.3 | 0.3 | 0.3 |  |  |  |
| RR | 1.2 | 1.3 | 1.4 | 1.5 | 1.6 | 1.7 | 1.8 |  |  |  |
| Alpha | Power | Power | Power | Power | Power | Power | Power |  |  |  |
| 0.1 | 0.280 | 0.419 | 0.563 | 0.694 | 0.799 | 0.876 | 0.928 |  |  |  |
| 0.05 | 0.183 | 0.301 | 0.438 | 0.576 | 0.700 | 0.800 | 0.875 |  |  |  |
| 0.01 | 0.064 | 0.127 | 0.220 | 0.336 | 0.464 | 0.590 | 0.703 |  |  |  |
| 0.005 | 0.039 | 0.085 | 0.157 | 0.256 | 0.374 | 0.499 | 0.619 |  |  |  |
| 0.001 | 0.012 | 0.032 | 0.068 | 0.127 | 0.210 | 0.313 | 0.428 |  |  |  |
|  |  |  |  |  |  |  |  |  |  |  |
| **Prevalence** | 0.25 | 0.25 | 0.25 | 0.25 | 0.25 | 0.25 | 0.25 | 0.25 | 0.25 | 0.25 |
| **RR** | 1.2 | 1.3 | 1.4 | 1.5 | 1.6 | 1.7 | 1.8 | 1.9 | 2 | 2.1 |
| **Alpha** | Power | Power | Power | Power | Power | Power | Power | Power | Power | Power |
| 0.1 | 0.257 | 0.380 | 0.511 | 0.636 | 0.744 | 0.828 | 0.890 | 0.933 | 0.960 | 0.977 |
| 0.05 | 0.165 | 0.266 | 0.387 | 0.513 | 0.633 | 0.737 | 0.819 | 0.881 | 0.925 | 0.954 |
| 0.01 | 0.055 | 0.107 | 0.183 | 0.280 | 0.391 | 0.507 | 0.617 | 0.714 | 0.795 | 0.858 |
| 0.005 | 0.033 | 0.070 | 0.128 | 0.208 | 0.306 | 0.415 | 0.526 | 0.631 | 0.724 | 0.800 |
| 0.001 | 0.010 | 0.025 | 0.052 | 0.097 | 0.161 | 0.243 | 0.338 | 0.441 | 0.544 | 0.640 |
|  |  |  |  |  |  |  |  |  |  |  |
| ***maf=0.06*** | 0.25 | 0.25 | 0.25 | 0.25 | 0.25 | 0.25 | 0.25 | 0.25 |  |  |
| **RR** | 1.2 | 1.3 | 1.4 | 1.5 | 1.6 | 1.7 | 1.8 | 1.9 |  |  |
| **Alpha** | Power | Power | Power | Power | Power | Power | Power | Power |  |  |
| 0.1 | 0.297 | 0.438 | 0.581 | 0.708 | 0.809 | 0.882 | 0.931 | 0.961 |  |  |
| 0.05 | 0.197 | 0.318 | 0.456 | 0.592 | 0.712 | 0.808 | 0.879 | 0.927 |  |  |
| 0.01 | 0.070 | 0.138 | 0.233 | 0.351 | 0.478 | 0.601 | 0.710 | 0.799 |  |  |
| 0.005 | 0.044 | 0.093 | 0.169 | 0.269 | 0.387 | 0.510 | 0.627 | 0.729 |  |  |
| 0.001 | 0.014 | 0.035 | 0.074 | 0.136 | 0.220 | 0.323 | 0.436 | 0.550 |  |  |
|  |  |  |  |  |  |  |  |  |  |  |
| ***maf=0.07*** | 0.25 | 0.25 | 0.25 | 0.25 | 0.25 | 0.25 | 0.25 | 0.25 |  |  |
| **RR** | 1.2 | 1.3 | 1.4 | 1.5 | 1.6 | 1.7 | 1.8 | 1.9 |  |  |
| **Alpha** | Power | Power | Power | Power | Power | Power | Power | Power |  |  |
| 0.1 |  |  |  | 0.768 | 0.859 | 0.920 | 0.957 | 0.978 |  |  |
| 0.05 |  |  |  | 0.661 | 0.777 | 0.862 | 0.919 | 0.955 |  |  |
| 0.01 |  |  |  | 0.421 | 0.558 | 0.682 | 0.784 | 0.861 |  |  |
| 0.005 |  |  |  | 0.180 | 0.284 | 0.405 | 0.528 | 0.644 |  |  |
| 0.001 |  |  |  | 0.333 | 0.466 | 0.596 | 0.710 | 0.803 |  |  |
|  |  |  |  |  |  |  |  |  |  |  |
| ***maf=0.08*** | 0.25 | 0.25 | 0.25 | 0.25 | 0.25 | 0.25 | 0.25 | 0.25 |  |  |
| RR | 1.2 | 1.3 | 1.4 | 1.5 | 1.6 | 1.7 | 1.8 | 1.9 |  |  |
| **Alpha** | Power | Power | Power | Power | Power | Power | Power | Power |  |  |
| 0.1 |  |  |  | 0.817 | 0.896 | 0.945 | 0.973 |  |  |  |
| 0.05 |  |  |  | 0.722 | 0.828 | 0.901 | 0.946 |  |  |  |
| 0.01 |  |  |  | 0.489 | 0.630 | 0.749 | 0.840 |  |  |  |
| 0.005 |  |  |  | 0.229 | 0.229 | 0.483 | 0.611 |  |  |  |
| 0.001 |  |  |  | 0.398 | 0.398 | 0.670 | 0.778 |  |  |  |
|  |  |  |  |  |  |  |  |  |  |  |
| ***maf=0.09*** | 0.25 | 0.25 | 0.25 | 0.25 | 0.25 | 0.25 |  |  |  |  |
| **RR** | 1.2 | 1.3 | 1.4 | 1.5 | 1.6 | 1.7 |  |  |  |  |
| **Alpha** | Power | Power | Power | Power | Power | Power |  |  |  |  |
| 0.1 |  |  |  | 0.856 | 0.924 | 0.963 |  |  |  |  |
| 0.05 |  |  |  | 0.773 | 0.869 | 0.929 |  |  |  |  |
| 0.01 |  |  |  | 0.553 | 0.693 | 0.804 |  |  |  |  |
| 0.005 |  |  |  | 0.281 | 0.417 | 0.557 |  |  |  |  |
| 0.001 |  |  |  | 0.461 | 0.608 | 0.734 |  |  |  |  |
|  |  |  |  |  |  |  |  |  |  |  |
| ***maf=0.10*** | 0.25 | 0.25 | 0.25 | 0.25 | 0.25 | 0.25 |  |  |  |  |
| **RR** | 1.2 | 1.3 | 1.4 | 1.5 | 1.6 | 1.7 |  |  |  |  |
| **Alpha** | Power | Power | Power | Power | Power | Power |  |  |  |  |
| 0.1 |  |  | 0.792 | 0.888 | 0.945 | 0.975 |  |  |  |  |
| 0.05 |  |  | 0.691 | 0.817 | 0.900 | 0.950 |  |  |  |  |
| 0.01 |  |  | 0.454 | 0.613 | 0.748 | 0.848 |  |  |  |  |
| 0.005 |  |  | 0.203 | 0.335 | 0.482 | 0.623 |  |  |  |  |
| 0.001 |  |  | 0.364 | 0.522 | 0.669 | 0.787 |  |  |  |  |
