# Supplementary material for: Increased rate of sporadic and recurrent rare genic copy number variants in Parkinson's disease among Ashkenazi Jews
Source: Mol Genet Genomic Med. 2013 Jun 7;1(3):142–54. doi: 10.1002/mgg3.18 (PMC3782064; doi:10.1002/mgg3.18)
Supplement: Supplementary file 6 [file mgg30001-0142-SD6.docx]

**Supplementary Table 1 Steps in the CNV QC process**

|  | | Filters/QC | Exclude CNV | Total | | | Cases | | | Controls | | |
| --- | --- | --- | --- | --- | --- | --- | --- | --- | --- | --- | --- | --- |
|  |  |  |  | IDs | CNV | Ave.CNV/ID | IDs | CNV | Ave.CNV/ID | IDs | CNV | Ave.CNV/ID |
| Clean CNV | Original | Num. raw CNV |  | 446 | 96220 | 215.74 | 268 | 65303 | 243.67 | 178 | 30917 | 173.69 |
|  | Step* | Sample and CNV QC |  |  |  |  |  |  |  |  |  |  |
|  | 1 | 11 sample_LRR_SD>0.27 | -3111 | -11 |  |  | -4 |  |  | -7 |  |  |
|  | 2 | CNV conf<10 | -5838 |  |  |  |  |  |  |  |  |  |
|  | 3 | CNV numsnp<5 | -3478 |  |  |  |  |  |  |  |  |  |
|  | 4 | CNV cent-tel-immu region | -951 |  |  |  |  |  |  |  |  |  |
|  | 5 | CNV length<100Kb | **-93462** | 409 | 1804 |  |  |  |  |  |  |  |
|  | 6 | Remove 3 excess num. (mean+3stdev=26) | -201 | 409 | 1603 |  |  |  |  | -3 |  |  |
|  | **Samples with CNV** | | | **406** | **1603** | **3.95** | **248** | **988** | **3.98** | **158** | **615** | **3.89** |
| **Passed QC sample** | | | | **432** |  |  | **264** |  |  | **168** |  |  |
| Rare CNV | 7 | 50%overlap | -344 |  | 1259 |  |  |  |  |  |  |  |
|  | 8 | Freq.>1% | -273 |  | **986** |  |  |  |  |  |  |  |
|  | **Samples with CNV** | | | **342** | 986 | 2.88 | **203** | 600 | 2.96 | **139** | 386 | 2.78 |

*filter1,2,3,4,5 exclude filter overlap part of CNV, total removed 94416 CNV
