# Supplementary material for: Increased rate of sporadic and recurrent rare genic copy number variants in Parkinson's disease among Ashkenazi Jews
Source: Mol Genet Genomic Med. 2013 Jun 7;1(3):142–54. doi: 10.1002/mgg3.18 (PMC3782064; doi:10.1002/mgg3.18)
Supplement: Supplementary file 7 [file mgg30001-0142-SD7.docx]

**Supplementary Table 3. List of Primers and Probes used for qPCR validation of CNVs**

**3a) Taqman Assays**

| CNV nearby gene | CNV left | CNV | CNV right |
| --- | --- | --- | --- |
| *CFH* | Hs03356469_cn | cfh3 | Hs03374552_cn |
| *SDK1* | Hs03650266_cn | Hs03628200_cn | Hs03638078_cn |
| *DLC1* | Hs00325218_cn | Hs01245954_cn | Hs03271306_cn |
| *PPYR1* | Hs03757398_cn | Hs00733699_cn | ppr1-r-cc6rm6k |
| *CYP2E1* | cyp2e1-l | Hs03765145_cn | cyp2e1r3 |
| *SYCE1* | cyp2e1-l | Hs00787229_cn | cyp2e1r3 |
| *BCHE* | Hs06657620_cn | Hs00309435_cn | Hs03374552_cn |
| *OVOS2* | Hs04404518_cn | Hs03823735_cn | Hs03812727_cn |

**Taqman Assays were designed to validate CNVs overlapping a nearby gene(s) and flanking regions (left and right).**

**3b) Custom Assays**

| Custom assay | CNV sequence position | Taqman Primers | |
| --- | --- | --- | --- |
| Cfh3 | chr1:196,747,769-196,823,175 | Forward | GTCAATAATATATCTCATCCGATACATGAATTCTAACT |
|  |  | Reverse | TCTTGAGTTTTGTGGCATTTTGTTTGT |
|  |  | Probe | TTCGATGGCATGCATTCTTACC |
| ppr1-r-cc6rm6k | chr10:49,199,973-49,199,996 | Forward | AGGCCCCACCCAGACA |
|  |  | Reverse | CAGCCCAAATATCTGCCAGTGA |
|  |  | Probe | TTGTGTAATTCCCCAGAGCAGGCC |
| Cyp2e1-1 | chr10:135,238,888-135,238,952 | Forward | GGTGAGACGGACAACAGTGTTTT |
|  |  | Reverse | GGTGAGACGGACAACAGTGTTTT |
|  |  | Probe | AAGAGCTGGAAAGAACATGTATTTG |
| cyp2er3 | chr10:135,386,289-135,386,314 | Forward | GTAAAAGACCTCTGTGGAGAACATCA |
|  |  | Reverse | TGTGGCAAACGGTCACCTT |
|  |  | Probe | CACAATATCCTGTGCATCCTCC |

**3c) Roche Universal probe library probe and primers for *SGCZ* Assay**

| *SGCZ* region | position (hg18) | Probe UPL# | Forward Primer | Reverse Primer |
| --- | --- | --- | --- | --- |
| 800K Left Flank | chr8:12953668-12955668 | 9 | CATTAAGTACATTTCCATTGTTGTGC | GGCTTGGGTTGTTGAAAAAG |
| 400K Left Flank | chr8:13353668-13355668 | 3 | TGCTGCAACAAATATGGGATT | AAAACAGAGCTACCATATGATCCA |
| 200K Left Flank | chr8:13553668-13555668 | 29 | TTCCAATTTTGCATATCCTTTCT | CATGTTTCTGTGCCCACTCA |
| 150K Left Flank | chr8:13593668-13595668 | 13 | CGTTTTTCCCTGTTCTTGCT | TCGCCACTGTCATTCAACAT |
| 100k Left Flank | chr8:13643668-13645668 | 55 | TGGGGTTTTCTTCTCATGTGT | GCAGCTGTTCCTTAAACCATGT |
| 10k Left Flank | chr8:13733668-13735668 | 33 | TGGCTATTTTTGTTCCCTTGTC | TCTACAGTTTCAATAAAATCCCTCAG |
| *SGCZ* Region 1 | chr8:13835534-13837534 | 18 | TCTCTTGTCTTGATAAATTGGCTCT | TGGATGAAATTTGTAACCTCTCAA |
| *SGCZ* Region 2 | chr8:14065705-14067705 | 69 | ACTGGGCCTTGAAGGTGAAT | ACTGCTGGCCATTATCTCAAA |
| *SGCZ* Region 3 | chr8:14375704-14377704 | 27 | TGGGGAAAGTTATAGGGGAAA | TTGCAAGTCACTCTTCCCAGT |
| *SGCZ* Region 4 | chr8:14626975-14628975 | 6 | TTCCATTGTGTATATAGACCACCTTTA | CATGTTTATTGCAACATTCTACACA |
| 10k Right Flank | chr8:14711986-14713986 | 55 | GGAGAACAGCCATTAAAAGAGTACA | CACGGCAGTGCGTCTAATAGT |
| 100k Right Flank | chr8:14801986-14803986 | 3 | GCACAGAAATCAACCAACGA | GGACCCAGCGGTATAGTTGA |
| Coagulation Factor VII (F8) | chrX:153810856-153812856 | 69 | GCTTGGTTTGATTTCCCAAG | GAACAAAGAAACACACAGCTCATT |
| Albumin (ALB) (reference) | chr4:74495691-74497691 | 77 | GCTTTCGTCTGTCCTATCTTCAA | ATAGCCAGGTTAAAAACAGAAAGG |

Primers used to detect the left flank of SGCZ gene, the SGCZ gene and right flank of SGCZ gene and two reference primers.
