# Supplementary material for: Increased rate of sporadic and recurrent rare genic copy number variants in Parkinson's disease among Ashkenazi Jews
Source: Mol Genet Genomic Med. 2013 Jun 7;1(3):142–54. doi: 10.1002/mgg3.18 (PMC3782064; doi:10.1002/mgg3.18)
Supplement: Supplementary file 8 [file mgg30001-0142-SD8.docx]

**Supplementary Table 4 Global rare CNV burden analysis and Gene counts**

| Type | Classification | Case CNV(n) | Control CNV(n) | Total CNV(n) | P | Case/ctrl ratio | Baseline rate (ctrl) |
| --- | --- | --- | --- | --- | --- | --- | --- |
| ALL | ALL | 600 | 386 | 986 | 0.535 | 0.99 | 4.30 |
| Deletions | ALL | 245 | 143 | 388 | 0.455 | 1.04 | 1.78 |
| Duplications | ALL | 355 | 243 | 598 | 0.613 | 0.96 | 2.52 |
| CNV frequency |  |  |  |  |  |  |  |
| ALL | 1x | 114 | 61 | 175 | 0.386 | 1.17 | 0.89 |
|  | 2-6x | 411 | 289 | 700 | 0.805 | 0.88 | 3.08 |
| Deletions only | 1x | 62 | 32 | 94 | 0.688 | 0.74 | 0.63 |
|  | 2-6x | 141 | 90 | 231 | 0.578 | 0.96 | 1.01 |
| Duplications only | 1x | 70 | 35 | 105 | 0.131 | 2.13 | 0.31 |
|  | 2-6x | 243 | 178 | 421 | 0.717 | 0.91 | 1.71 |
| CNV size |  |  |  |  |  |  |  |
| ALL | 100-500 kb | 585 | 372 | 957 | 0.388 | 1.05 | 3.84 |
|  | ≥500kb | 15 | 14 | 29 | 0.735 | 0.31 | 0.46 |
|  | ≥1 Mb | 5 | 2 | 7 | 0.290 | 2.13 | 0.05 |
| Deletions only | 100-500 kb | 241 | 142 | 383 | 0.283 | 1.17 | 1.49 |
|  | ≥500kb | 4 | 1 | 5 | 0.656 | 0.39 | 0.30 |
|  | ≥1 Mb | 1 | 0 | 1 | 0.602 | N/A | 0.00 |
| Duplications only | 100-500 kb | 344 | 230 | 574 | 0.574 | 0.97 | 2.36 |
|  | ≥500kb | 11 | 13 | 24 | 0.720 | 0.70 | 0.16 |
|  | ≥1 Mb | 4 | 2 | 6 | 0.485 | 1.31 | 0.05 |

Supplementary Table 4 shows gene count in cases versus controls. Total burden for genes intersected by CNVs in AJ cases (n=261) compared to controls (n=171). Gene coordinates were defined by the RefSeq boundaries plus a 20-kb region on either side. All genomic analyses used NCBI Build 36. Genome-wide P-values were estimated in 100,000 permutations (one-sided) for global case–control differences in CNV rate and size. Analyses were further stratified according to CNV type (‘deletions only’ and ‘duplications only’) and frequency (single occurrences (1x) or CNVs observed two to six times in the total sample (2–6x)).Note that for the two lower-frequency groups, the ‘deletions only’ and ‘duplications only’ counts are not expected to sum to the ‘all’ count.
