# Supplementary material for: Increased rate of sporadic and recurrent rare genic copy number variants in Parkinson's disease among Ashkenazi Jews
Source: Mol Genet Genomic Med. 2013 Jun 7;1(3):142–54. doi: 10.1002/mgg3.18 (PMC3782064; doi:10.1002/mgg3.18)
Supplement: Supplementary file 9 [file mgg30001-0142-SD9.pptx]

## Slide 1
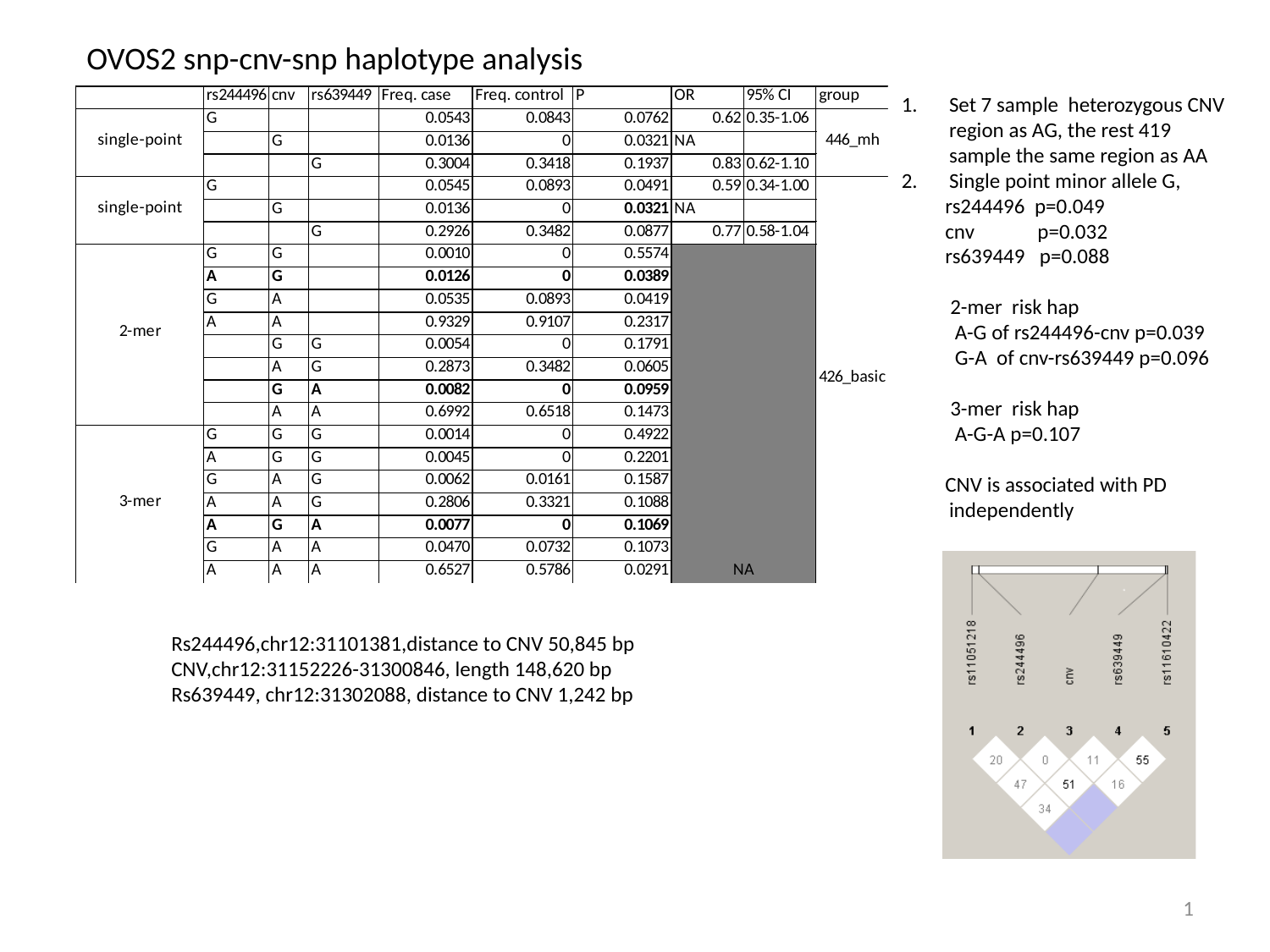

OVOS2 snp-cnv-snp haplotype analysis
Set 7 sample heterozygous CNV region as AG, the rest 419 sample the same region as AA
Single point minor allele G,
 rs244496 p=0.049
 cnv p=0.032
 rs639449 p=0.088
 2-mer risk hap
 A-G of rs244496-cnv p=0.039
 G-A of cnv-rs639449 p=0.096
 3-mer risk hap
 A-G-A p=0.107
 CNV is associated with PD independently
Rs244496,chr12:31101381,distance to CNV 50,845 bp
CNV,chr12:31152226-31300846, length 148,620 bp
Rs639449, chr12:31302088, distance to CNV 1,242 bp
1
